# Are Endothelial Progenitor Cells the Real Solution for Cardiovascular Diseases? Focus on Controversies and Perspectives

**DOI:** 10.1155/2015/835934

**Published:** 2015-10-05

**Authors:** Carmela R. Balistreri, Silvio Buffa, Calogera Pisano, Domenico Lio, Giovanni Ruvolo, Giuseppe Mazzesi

**Affiliations:** ^1^Department of Pathobiology and Medical Biotechnologies, University of Palermo, 90134 Palermo, Italy; ^2^Unit of Cardiac Surgery, Department of Surgery and Oncology, University of Palermo, 90134 Palermo, Italy; ^3^Department of General and Specialist Surgery, University of Rome “Sapienza”, 00161 Rome, Italy

## Abstract

Advanced knowledge in the field of stem cell biology and their ability to provide a cue for counteracting several diseases are leading numerous researchers to focus their attention on “regenerative medicine” as possible solutions for cardiovascular diseases (CVDs). However, the lack of consistent evidence in this arena has hampered the clinical application. The same condition affects the research on endothelial progenitor cells (EPCs), creating more confusion than comprehension. In this review, this aspect is discussed with particular emphasis. In particular, we describe biology and physiology of EPCs, outline their clinical relevance as both new predictive, diagnostic, and prognostic CVD biomarkers and therapeutic agents, discuss advantages, disadvantages, and conflicting data about their use as possible solutions for vascular impairment and clinical applications, and finally underline a very crucial aspect of EPCs “characterization and definition,” which seems to be the real cause of large heterogeneity existing in literature data on this topic.

## 1. Introduction

The most important determinant of cardiovascular health is person's age [1]. By 2030, approximately 20% of the population will be aged 65 or older [2]. In this age group, cardiovascular diseases (CVDs) will result in 40% of all deaths and rank as the leading cause [2]. Furthermore, the cost to treat CVDs will triple in that time [3]. Of consequence, urgent interventions both in preventive measures and biomedicine research are imperative. In the last years, some progresses have been realized. For example, primordial prevention based on healthful lifestyle (i.e., Mediterranean diet, lifestyle, and physical activity) has been proposed as preferred preventive's method to lower cardiovascular risk [4]. Advances have been achieved through percutaneous coronary intervention and coronary artery bypass grafting in management of coronary artery diseases, having higher prevalence and incidence in the world [5, 6]. Despite these efforts, there are no effective solutions until now. In addition, numerous gaps still remain between knowledge of precise CVD cellular and molecular mechanisms and identification of disease pathways to use as appropriate biomarkers and targets for new and more efficient therapeutic treatments, that is, personalized therapies.

Biomedical community is pursuing new ways in trying to face this imposing challenge. In particular, the latest discoveries and advanced knowledge in the fields of stem cell biology and their ability to provide a cue for counteracting several diseases are leading numerous researchers to focus their attention on “*regenerative medicine*” as possible solutions for CVDs [7]. However, the lack of consistent evidence in this arena has hampered the clinical application [8]. The same condition affects the research on endothelial progenitor cells (EPCs), creating more confusion than comprehension. In this review, this aspect is discussed with particular emphasis. In particular, we describe biology and physiology of EPCs, outline their clinical relevance as both new predictive, diagnostic and prognostic CVD biomarkers, and therapeutic agents, discuss advantages, disadvantages, and conflicting data about their use as possible solutions for vascular impairment and clinical applications, and finally underline a very crucial aspect of EPCs “*characterization and definition,*” which seems to be the real cause of large heterogeneity existing in literature data on this topic.

## 2. Recent Efforts of Biomedical Research in Cardiovascular Repair: EPC Cells as Promising Candidates

Actually, the principal purpose of scientific community is to improve life quality and reduce and/or retard CVD onset and progression, even if it appears to be very ambitious. Its realization seems to be difficult for different reasons. Firstly, CVDs have a very complex pathophysiology orchestrated by mechanisms not completely clear and articulated in multistep clinical events. Another limiting factor is CVD progression generally assumed as irreversible and one-directional [9]. However, a small reverse probability has been recently suggested for each step. Accordingly, some individuals, even in the presence of potent risk factors, remain sheltered from consequences of cardiovascular alterations. The potential reason has been attributed to substantial ability to have an efficient cardiovascular self-repair, which appears to be prevalently modulated by genetic background and environmental factors [9]. As result, the interest on cardiovascular repair is increasing. It has led to evidence that three major processes drive it: (i) replacement (tissue transplant), (ii) rejuvenation or restoration (activation of resident or not stem and progenitor cells via autocrine, paracrine, or endocrine mechanisms; modulation of apoptosis, inflammation, angiogenesis, or metabolism), and (iii) regeneration (progenitor or stem cell engraftment forming differentiated cardiovascular cells) [10]. The three different entities may singularly function or be interlinked [10]. However, their mechanisms remain to be determined. Furthermore, in the regeneration, hematopoietic stem and progenitor cells (HSCs and HPCs) seem to have a crucial role. HSCs and HPCs are, indeed, becoming the potential therapy's agents for improving reparatory mechanisms in the heart and vascular system. Many studies have investigated their role in different CVDs, such as acute coronary syndromes, stroke, limb ischemia, and cardiac nonischemic injury. Discordant results have been obtained [11]. Thus, their real contribution is until now uncertain. However, it has been observed that cardiovascular risk factors induce impairment in their circulating levels and function. In contrast, physical exercise and statins mediate their improvement [11]. Of note, it also is their contribution in physiological endothelial and cardiac renewal, as observed in healthy subjects [11]. However, the weight of these observations is remarkably influenced by an essential limitation. HSCs and HPCs have been identified only as CD34^+^ cells. Thus, the validity of these results needs to be confirmed.

Among the HSCs and HPCs, EPCs are the most widely studied adult human progenitor cell subpopulation up to now. Here, we report a summary of literature data on biological features of EPC cells.

## 3. Biological EPC Features

### 3.1. EPC Origins and Sources

EPC's discovery occurred in 1997 by Asahara and colleagues, which questioned the paradigm of angiogenesis and vasculogenesis in adult, by identifying H-precursor cells, defined as EPC cells able to differentiate into an endothelial phenotype* ex vivo* [12]. From then, a plethora of evidence supports EPC existence, origins, and contribution in new blood vessel formation [13]. EPCs have, indeed, capacity to proliferate, migrate, and differentiate into mature endothelial cells (ECs). In 2004, Urbich and Dimmeler defined EPCs using three biological parameters: (1) to be nonendothelial cell, but having capacity to give rise to ECs and (2) to show clonal ability to multiply, (3) and stemness characteristics [14].

Concerning their origin and sources, they have been object of a strong debate for different years. Actually, EPCs can be divided into two categories: H-EPCs and non-H-EPCs [13, 15, 16]. Here, we try to clarify this relevant and delicate aspect. We also point EPC origin from cord blood, as another relevant source.

#### 3.1.1. H-EPCs

HSCs (expressing the classical CD34 marker or more immature CD133 marker) are the principal EPC source (see Table 1). They are maintained within bone marrow (BM) stem cell niches and released upon induced mobilization (see below), as firstly demonstrated by Asahara and colleagues [12]. This initial discovery has led to define EPCs as CD34^+^ or CD133^+^ cells. HSC contribution to neovascularization has been initially evaluated in animal models [16]. The promising results obtained have led to several clinical studies on progenitor cell therapy (in humans, see below) [13, 15, 16].

However, other BM-stem cells can generate EPCs, including BM-myeloid cells and BM-mesenchymal stem cells (MSC) (see Table 1). BM-myeloid cells are also mobilized from BM and derive from HSCs. Schmeisser and colleagues evidenced that CD14^+^/CD34^−^ myeloid cells can coexpress endothelial markers and form tubelike structure* ex vivo* [17]. Thus, BM-myeloid cells within peripheral blood can differentiate into endothelial lineage with a lower proliferative capacity than HSCs or cord blood derived EPCs [13]. Certainly, additional studies are necessary to determine differences in incorporation and particularly to clear the long-fate of HSCs versus monocyte derived cells [13, 15, 16].

BM also contains MSCs, which are stromal cells having ability to self-renew and also exhibit multilineage differentiation into both mesenchymal and nonmesenchymal lineages. BM-MSCs can differentiate into ECs and improve neovascularization, as demonstrated by* in vitro* studies. In addition, BM-MSCs have been also isolated from peripheral blood. This has opened the question on possibility of their mobilization in case of ischemia and their contribution to endogenous cardiovascular repair [13, 15, 16]. Further studies are, certainly, necessary for clarifying this question.

#### 3.1.2. Non-H-EPCs

Other cell populations from other sources (i.e., adipose tissue, blood vessel wall, liver, intestine, spleen, and kidney) can give rise to EPCs [13, 15, 16] (see Table 1).

Adipose tissue represents an alternative source of autologous adult stem cells, which can be obtained in large quantities under local anaesthesia and with minimal discomfort. Human lipoaspirate contains stem cells able to differentiate into several lineages. Furthermore, it has been also observed that isolated-tissue-derived, cultured, and stromal-vascular CD34^−^CD31^−^ cell fractions can differentiate into ECs and promote angiogenesis [13, 15, 16].

Furthermore, MSCs, originally identified in BM, have been also detected in many other tissues, such as adipose tissue. They are able to differentiate into EC mature cells in an appropriate microenvironmental. In addition, they show ability to modulate immune responses. This leads to consider them as more attractive candidates for regenerative medicine. Allogeneic transplant of these cells is feasible without a substantial risk of immune rejection. MSCs secrete various immunomodulatory molecules which provide a regenerative microenvironment for a variety of injured tissues or organs to limit the damage and to increase self-regulated tissue regeneration. Autologous/allogeneic MSCs delivered via the bloodstream augment the titers of MSCs that are drawn to sites of tissue injury and can accelerate the tissue repair process [17, 18]. Recently, it has been also discovered that MSCs also derive from a perivascular location, where they reside as pericytes or adventitial cells. This finding has generated some momentum in the field of adult stem cell research and provided some insights into the developmental origins of these much exploited but little understood cells. It is now evident that the perivasculature represents MSC niche* in vivo*, where local cues coordinate the transition to progenitor and mature cell phenotypes. Here, MSCs can stabilize blood vessels and contribute to tissue and immune system homeostasis under physiological conditions and assume a more active role in tissue repair in response to injury. The establishment of a perivascular compartment as the MSC niche provides a basis for the rational design of additional* in vivo* therapeutic approaches [19, 20].

#### 3.1.3. Cord Blood EPCs

A rich EPC source also is cord blood (see Table 1). Cord blood contains higher numbers of CD133^+^ and CD34^+^ cells compared with peripheral blood from adults CD133^+^/CD34^+^ cells [13, 15, 16]. In addition, a higher proliferation capacity and high levels of telomerase have been evidenced in cord blood derived EPCs [13, 15, 16]. These characteristics are typical of stem cells and very low or absent in other progenitor cell populations.

### 3.2. EPC Recruitment and Mobilization from BM, Their Migration, and Adhesion to Injured Vessel Wall

The EPC related formation of new blood vessels includes multiple steps comprising mobilization, migration, adhesion, and differentiation [21]. The mobilization of EPCs from BM into the peripheral circulation is the crucial step for these cells to participate in postnatal vasculogenesis. The precise mechanism of EPC mobilization is not entirely elucidated and it is still under investigation. It has been demonstrated that these cells are quiescent and tethered by integrins to stromal cells in a microenvironment within the BM. They can be converted into functional cells and released from the stem cell niche in response to various special cytokines and factors [21]. Mature ECs represent the crucial players in initiating H-EPC mediated vasculogenesis, by releasing attracting EPC factors under shear stress and hypoxia [13, 15, 16, 21, 22]. In the case of vascular occlusion, it has been observed that ECs seem to sense altered (low or oscillatory) shear stress and consequently improve prooxidant enzyme expression, mediated principally by the most crucial transcription factor, Nuclear factor kappa-light-chain-enhancer of activated B cells (NF-*κβ*) [13, 15, 16, 21, 22]. In case of hypoxia, several signaling pathways on ECs are stimulated. They particularly induce activation of hypoxia-inducible transcription factor (HIF) [22]. As result, different growth factors, cytokines, and chemokines are released mediating H-EPC mobilization. Another crucial and specific factor associated with EPC mobilization from BM is nitric oxide (NO), as demonstrated in endothelial NO synthase (eNOs)^−/−^ mice [23] (see below) (Table 2).

Furthermore, several types of chemokines are involved in EPC mobilization, such as stromal cell derived factor-1 (SDF-1), angiopoietin (Ang-1), and, probably the most important of all, vascular endothelial growth factor (VEGF) [21]. VEGF seems to determine a rapid EPC and HSC mobilization, as evidenced by Fox and colleagues in burned patients [21]. This last aspect has also consented to detect EPCs in peripheral blood as VEGFR2^+^ or KDR^+^ (see below and Table 5). After their homing, EPCs can release VEGF themselves and create a local angiogenetic environment. Recently, Li and colleagues reported that SDF-1 and VEGF mediate EPC mobilization, through their interaction with their respective receptors (C-X-C chemokine receptor type 4 (CXCR4) and VEGFR2). This interaction determines the production of NO through the activation of eNOs. NO can stimulate metalloproteinase-9, which results in the release of sKitL from the stromal cell membrane-bound kit ligand (mKitL). Protooncogene c-kit (c-kit) expressed by EPCs contributes to the retention of EPCs within the BM niches. C-Kit is also the receptor for sKitL and can be released from BM in response to binding to sKitL, resulting in mobilization of c-Kit^+^ EPCs from the cell niche into circulation [21].

Other factors as erythropoietin (EPO) can mobilize EPCs [24]. Ang-1 seems to have a delayed and inhibitory effect, as evidenced in a unique study performed in 1999, where EPCs were defined as Tie^+^/Flk-1^+^/CD31^+^ cells [25].

Adhesion of EPC cells to injured vessel wall involves the interaction between glycoprotein ligand-1 (PSGL-1) expressed on EPCs and P-selectin expressed on platelets, as suggested by Li and colleagues [21]. Within minutes after vessel injury, platelets, indeed, aggregate on the exposed subendothelium. Adherent platelets express P-selectin on the surface and secrete high levels of SDF-1. In this process, circulating EPCs also upregulates PGSL-1 via the stimulation of SDF-1, which interact with their ligand P-selectin, thereby leading to EPC adhesion. Subsequently (within the next hours and days after endothelial disruption), apoptotic smooth muscle cells mainly contribute to SDF-1 release, which is required to sustain the process of vascular remodeling and repair [21].

### 3.3. Circulating EPC Levels and Their Alterations: Effects Mediated by Different Factors

**Figure 1:**
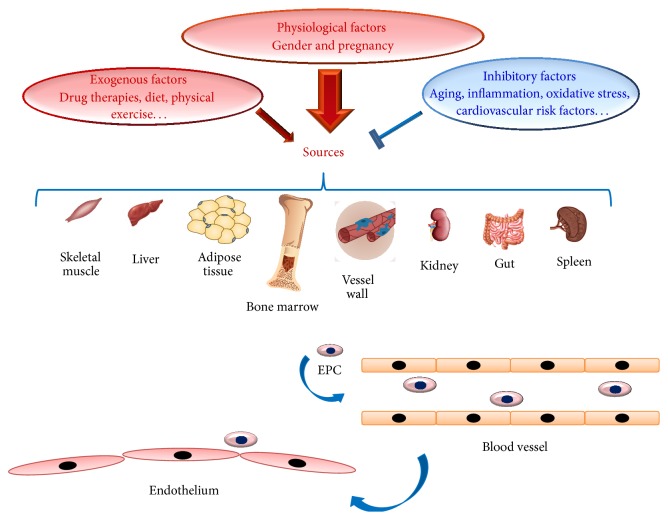
The several origins and sources of EPC cells.

Augmented or reduced circulating EPC levels, as well as their function, have been observed in a large number of studies. Several factors have been identified as possible causes (see Table 2 and Figure 1). Here, we describe them and their effects on EPC number and function.

#### 3.3.1. Unfavourable Factors Modulating Circulating EPC Levels

Different endogenous factors can also influence EPC levels (Table 2 and Figure 1). In particular, ageing has been associated with an altered EPC function and viability, by determining a decreased potentiality of endothelial repair [26, 27]. Recently, it has been suggested that age-related inflammation and oxidative stress modulate EPC bioactivity and determine dysfunction [28, 29]. In particular, increasing evidence indicates EPC mobilization in case of transient restricted inflammatory response. On the contrary, persistent or excessive inflammatory stimuli may have deleterious effects, by decreasing EPC circulating numbers [28, 29]. Functional EPC activity is significantly impaired in case of high inflammatory stimulation, as in heart failure. Mechanisms regulating this effect are still unclear. However, convincing evidence leads to suppose that prolonged exposure of BM to increased proinflammatory stimulation may determine EPC pool exhaustion. In this condition, a small EPC number, prevalently immature or dysfunctional, might be released. However, existing clinical evidence on association of inflammation with reduced EPC levels is largely circumstantial and observational [28, 29]. Thus, further clinical studies are required.

As mentioned above, oxidative stress may also play a crucial role in EPC mobilization from BM and functional bioactivity. ROS exert a direct cytotoxic effect on the vascular endothelium. Increased superoxide generation reduces EPC levels and impairs EPC function, as demonstrated by increased apoptosis and reduced EPC number after incubating with high levels of hydrogen peroxide (H_2_O_2_) [28, 29] (see Table 2).

An increasing body of evidence also suggests that cardiovascular risk factors (smoking, diabetes, hypertension, lipid disorders, abdominal obesity, metabolic syndrome, etc.) affect EPC number and proprieties [30] (see Table 2 and Figure 1).

Endocrine disorders, such as hyperparathyroidism and hypothyroidism, may also alter EPC levels (see Table 2) [31, 32].

#### 3.3.2. Physiological Factors Involved in Raising Circulating EPC Levels

An increased number of studies have demonstrated that physiological factors influence EPC circulating levels and function. Among physiological factors, gender appears to modulate EPC levels, as demonstrated by Fadini and colleagues [33]. Women have high EPC levels than men and oestrogens are the physiological factors significantly associated with these useful effects [31] (see Table 2 and Figure 1). In addition, pregnancy represents the physiological condition characterized by high EPC circulating levels [34].

#### 3.3.3. Drug Therapies, Nutrition Interventions, and Lifestyle Modifications as Strategies to Improve Circulating EPC Levels

Drug therapies can also influence EPC levels and function in a positive manner. They prevalently operate as anti-inflammatory and antioxidant factors. In 2014, Lee and Poh stressed the significant interaction between cardiovascular pharmacotherapies and improvement of EPC number and functions. In particular, they reported the effects observed in clinical studies on EPC number and function from patients with different CVDs and treated with different medications, including antihypertensive, cholesterol lowering, and antidiabetic medications [35] (see Table 2 and Figure 1).

Recently, a growing number of studies are also evidencing an improvement of EPC number and function related to nutrition interventions and lifestyle modifications (see Table 2 and Figure 1). In particular, some research groups are reporting that Mediterranean diet determines an increase in circulating EPC levels and function [36, 37]. Similarly, physical exercise seems to induce an improvement of circulating EPC levels (see Table 2 and Figure 1) [26]. This has been evidenced in both healthy subjects and patients affected by CVDs. Thus, even in patients, with diffuse atherosclerosis and multiple risk factors, reparative capacity dependent on circulating BM-derived EPC is retained and can be enhanced in a most physiological way [26].

## 4. EPC in Vascular Impairment and Their Clinical Relevance

**Figure 2:**
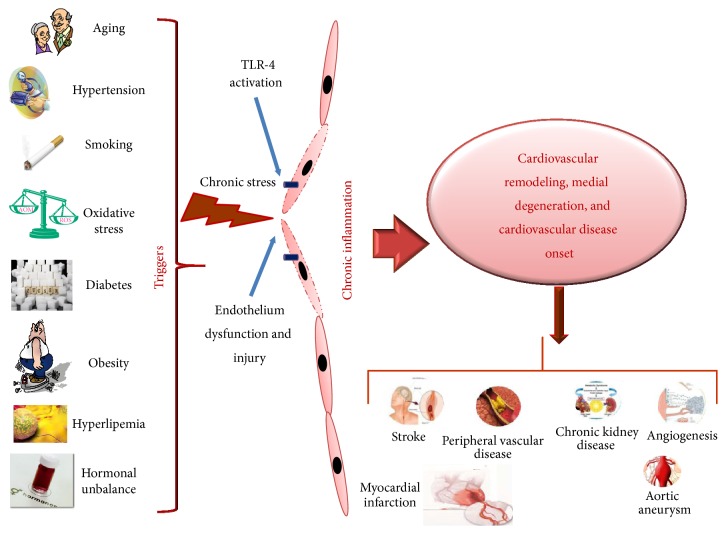
Endothelium dysfunction, injury, cardiovascular remodeling and onset of CVD diseases. Several factors (ageing, hypertension, oxidative stress, diabetes, hyperlipemia, obesity, and unbalance of hormones) by acting as triggers determine a chronic stress on endothelium of vascular wall and evocation of a chronic inflammatory response which cause endothelium dysfunction, injury, and cardiovascular remodeling and the onset of several CVDs.

The important role of ECs in maintaining of the entire vessel wall (of arteries or veins) homeostasis, as well as their recognized finite lifespan and continuous response to different triggers responsible of endothelium dysfunction and injury, is well recognized (see Figure 2) [38–40]. This has led to identify a system able to replace these cells. This system has been conventionally established and identified in mature ECs adjacent to regions of injury [38–40]. It has been speculated that, under influence of paracrine mediators released from the injured segments and/or loss of contact inhibition, ECs migrate and proliferate [38–40]. Today, it is recognized that mature ECs possess limited regenerative capacity [41–44]. The discovery of EPCs has opened this question. EPCs seem to be a real source of ECs in maintaining vascular homeostasis. Thus, they constitute a very reservoir of circulating cells, which could home to sites of injury, restore endothelium integrity, and consent a normal function. The contribution of EPCs to vascularization has been demonstrated in animal models and in humans (see below). Their crucial role in this process has cotemporally led to hypothesize that a reduction in EPC circulating number and/or alterations in their functions associated with different factors (as the above discussed) might have a remarkable impact on endothelium function and CVD onset and complications and consequently in the survival of CVD affected individuals [41–44]. Accordingly, growing evidence is underling the clinical relevance of EPCs as biomarkers of vascular function and cardiovascular risk in healthy individuals, as well as diagnostic and prognostic CVD biomarkers. In addition, several studies report how EPC can be used as therapeutic agents.

We below report data existing in literature about the possible clinical applications of these cells.

### 4.1. EPCs as Predictive CVD Biomarkers

The relationship between EPC circulating levels and cardiovascular risk might be of clinical relevance, and possible new recommendations and preventive CVD measures might be applied. Accordingly, in 2003, Hill and colleagues showed that the number of circulating EPCs represents a better predictor of vascular reactivity than conventional cardiovascular risk factors [45]. In addition, a significant correlation between* in vitro* EPC senescence and CVD risk profile has been also reported in donors. Thus, EPCs might be considered as an optimal biomarker for vascular function and cardiovascular risk. Certainly, ulterior studies are needed.

### 4.2. EPCs as Diagnostic and Prognostic CVD Biomarkers

Abnormalities in circulating EPC levels and function have been observed in a large number of studies on different CVDs. As result, EPCs have been suggested as diagnostic and prognostic CVD biomarkers [35, 40]. We report a summary of literature data in Table 1S (see Supplementary Materials available online at http://dx.doi.org/10.1155/2015/835934).

### 4.3. EPCs as Therapeutic Agents

Since the successful isolation of EPCs in 1997 [12], encouraging data have demonstrated EPC presence in the sites of vascular injury and ischemia. This has led to perform several preclinical studies in animal models (see Table 3). Promising findings have been obtained. In particular, a favorable improvement in left ventricular (LV) function in a rat model of myocardial infarction (MI) after intravenous injection of* ex vivo* expanded human CD34^+^ cells has been reported [46]. Furthermore, another study examined the effect of catheter-based intramyocardial transplantation in a swine model of MI, providing encouraging outcomes in favoring the application of EPCs as a potential cell therapy in clinical trials [47, 48]. In 2005, Naruse and colleagues carried out a study related to the therapeutic treatment of diabetic neuropathy by* in vivo* expanded human EPCs, using streptozocin-induced diabetic Nude rats [49]. They developed augmented conduction velocity and ameliorated blood flow of sciatic nerve. An increased number of microvessels were also observed on the site of EPC injection [49]. These results led to use this treatment for cerebrovascular disease [50]. An improvement of neurological functions was reported in chronic cerebral ischemic rats injected with CD34^+^ HSC cells, including EPCs [50] (see Table 3).

The ability of EPCs to expand in cultures under* in vitro* conditions raises another hesitant vision for their therapeutic use. Genetically modified and* ex vivo* expanded EPCs may become new promising agents, which will be able to appropriately rescue impaired neovascularization process under disease conditions. In Rhesus model,* ex vivo* CD34^+^ cell transfection with recombinant nonreplicative herpes virus vector and subsequent cell transplantation resulted in the expression of vector genes in angiogenic areas of skin autografts of rhesus macaques. Since CD34^+^ cells possess a natural angiogenic tropism to injured endothelium, they may serve as ideal candidates for the delivery of genes into areas of angiogenesis [51] (see Table 3).

These encouraging data have led to perform clinical trials in order to detect whether EPCs increase endothelial integrity and vascularisation at ischemia sites in patients with CVDs. Three different strategies have been principally used, as reported in Table 4. The first strategy consists in the* administration of granulocyte-colony stimulating factor (G-CSF)* in the order to determine the recruitment of the patient's own BM resident progenitors. Using this treatment, two preliminary studies demonstrated an increased LV function [52]. This certainly requires a confirmation in large studies. The second is* the intracoronary infusion of BM progenitor cells in patients with MI*. It demonstrated positive effects on LV function in three smaller studies [53–55]. Subsequently, two prospective large trials assessed significant LV function after 4–6 months of administration of BM progenitor cells. Other ten recent and large trials confirmed the successfulness and the safety of this procedure with a follow-up over 1.5 years [56, 57]. In addition, the intramyocardial and intracoronary administration has been recently suggested as a suitable strategy for treatment of patients with refractory angina [58]. The third strategy is more invasive and consists in* the direct injection of cells into target tissues* [59]. This treatment (and precisely* transepicardial or transendocardial injection of unfractioned BM cells*) has been performed in patients with diffuse coronary artery disease and intractable angina with no option of recanalisation. Ventricular function and physical capacity have been observed to increase, but the small sample size of these studies requires to be confirmed in larger studies [60–62] (see Table 3).

The treatment with* direct administration of EPCs* has been also effectuated in patients with chronic limb ischemia, demonstrating a reduced rate of limb's amputation at 3 years of follow-up [63, 64] (see Table 3).

Of special interest are the studies with* autologous cell therapy*. In line with this, the Yamamoto group performed an intramuscular injection of autologous BM-derived mononuclear cells containing 1% of CD34^+^ cells in patients with chronic limb ischemia [65]. They quantitatively evaluated the expression of EPCs and endothelial markers (i.e., CD133 and VE-cadherin) before the experiment and after the injection. Before investigation, the transcription of these molecules was undetectable. Autologous injection caused an elevation of EPC marker transcription. Thus, they concluded that autologous BM cells may be used in the therapy of patients with arterial diseases. A replication of these results was obtained by Lenk and colleagues [66]. Erbs and colleagues used this autologous treatment in patients who underwent recanalisation of chronic coronary total occlusion [67]. The autologous treatment with EPCs, expanded four days in endothelium growth medium, improved coronary endothelium function and wall motion abnormalities and had a benefit effect on the metabolism in the target area in patients with symptomatic coronary atherosclerosis [67] (see Table 3).

Despite of these promising data, EPC clinical application as exogenous or autologous cell therapy remains still unclear because of different reasons. We below discuss the limitations on EPC clinical applications.

## 5. Focus on Controversies and Perspectives about the EPC Clinical Use as Possible Solutions for Vascular Impairment and CVDs

Since their discovery, EPCs have been object of an intensive investigation and a plethora of clinical applications has been opened, as reported above. As result, EPCs have been suggested as potential predictive, diagnostic, and prognostic CVD biomarkers, as well as therapeutic agents. These efforts have encouraged the researchers in the vision to modulate the vasculogenesis process and consequently potentiate cardiovascular self-repair. However, the enthusiasm is actually dampened by a large number of critical viewpoints [68–72]. In particular, insights into EPC biology are leading several research groups to discuss on critical EPC aspects and to evidence the limitations. Thus, these perspectives reduce the large relevance and potentiality of these cells and cotemporally underline urgent necessity to move versus standardized and common criteria of research for EPC cells. This might reduce the heterogeneity of EPC literature data.

**Figure 3:**
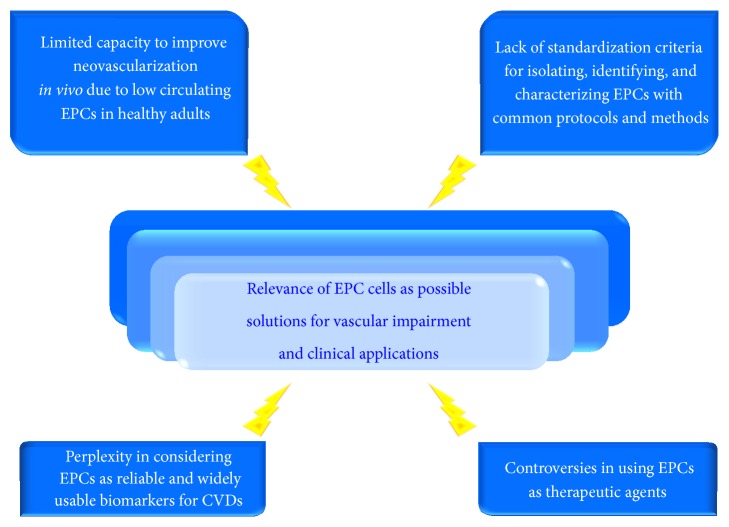
Critical aspects of the EPC relevance as possible solutions for vascular impairment and clinical applications. As reported in the figure and text (see Section 5), four critical aspects reduce the EPC potentiality as potential actors of endothelium repair, optimal CVD biomarkers, and therapy agents.

Here, we summarize the aspects of EPC cells principally discussed by scientific community (see Figure 3).

### 5.1. Real Capacity of EPCs Cells to Improve* In Vivo* Neovascularization

In healthy adults, EPC cells (as CD34^+^CD133^+^VEGFR2^+^ EPC cells) represent only 0.0001%–0.01% of peripheral blood mononuclear cells (PBMCs) [73]. These low percentages lead to question on their impact in pathological or physiological processes. Current evidence reports changes in EPC number and function in several CVDs (see Table 1S). However, different factors may influence levels and viability of EPC cells, including methodological approaches (i.e., the timing and ways of taking samples) [74], detection methods and their protocols, panel of antibodies used for their phenotypical evaluation, age of patients and their clinical conditions, and ethnicity of populations studied (see below).

### 5.2. EPCs as Vascular Healthy and CVD Biomarkers

It is current opinion that number and/or functionality of EPCs do not adequately describe CVD risk. This perplexity is due to inconsistent EPC definitions, different number of CVD risk factors in different patient populations studied, and the interaction of EPCs cells with other HPCs, inflammatory cells, and platelets.

### 5.3. EPCs as Therapeutic Agents

**Figure 4:**
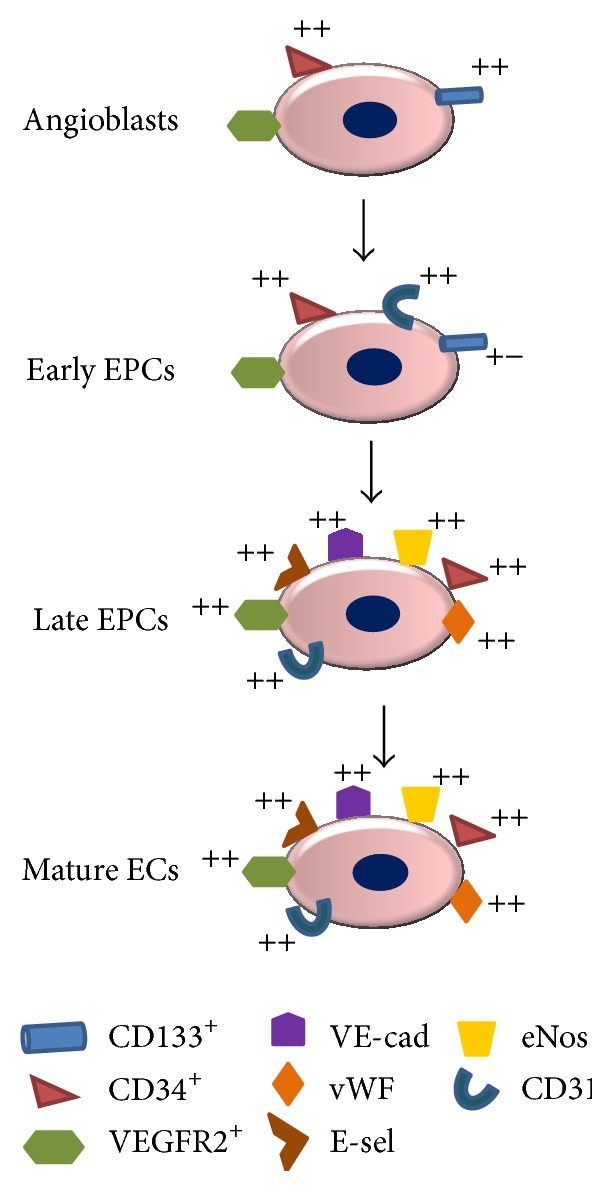
Angioblast differentiation into mature endothelial cells according to the schema proposed by Hristov and Weber, 2004 [75]. As illustrated in the figure, CD133^+^, CD34^+^, and VEGFR2^+^ (CD309^+^) angioblasts give rise to early EPCs expressing high intensity CD31, CD34, and CD309 markers which differentiate in late outgrowth endothelial cells (OEC)s, having high expression not only of CD34, CD309, and CD31 but also of vWF, E-selectin, VE-cadherin, and eNOs.

Available clinical studies of EPCs as therapeutic agents show beneficial results (as described above). However, their validity is limited by different factors: (a) the small number of patients enrolled in the major number of studies, their randomization not blinded, the involvement of few centres, (b) the exact phenotypic profile of cells used for the treatments which is always not indicated or missing, (c) the different administration ways and methods used, and (d) the safety and feasibility of the treatments not proved by long-term follow-up results. Teratoma formation, immunoreactivity, or arrhythmias may represent the adverse effects of these treatments. In addition, there are other limitations in the large-scale clinical use of EPCs. As the above mentioned, EPCs are relatively rare cells, and expansion of sufficient numbers of subpopulations from peripheral blood is hardly possible. Furthermore,* in vitro* enumeration of progenitor cells for a quantity sufficient for a therapeutic treatment is associated with changes in phenotype and differentiation and risk of cell senescence and it may require artificial cell preactivation or stimulation. The* in vitro* cultures consent the production of two subpopulations from CD133^+^/CD34^+^/CD309^+^ BM-hemangioblasts according to Hristov and Weber's schema [75], the early EPCs (eEPCs) and late EPCs (outgrowth endothelial cells, OECs), having different features (see Figure 4).

### 5.4. Lack of Standardized Criteria and Consensus for Defining, Characterizing, and Identifying EPCs with Well Established Surface Markers, Protocols, and Methods

EPCs cells have been largely described as CD34^+^CD133^+^VEGFR2^+^ cells [69]. However, other progenitor populations have been recently considered in EPC studies, that is, circulating angiogenic cells (CACs), circulating endothelial cells (CECs), circulating H-progenitor cells (CPCs), and circulating endothelial progenitors (CEPs), playing important roles in tissue neovascularization, but having diverse features [76]. CAC and CEP cells represent variable proportions of CD14^+^ monocyte cells having different angiogenic properties. Despite their lower* in vitro* proliferation than HSCs or cord stem cells, they seem to have a similar ability to increase neovascularization, as reported in experimental models [76]. This leads to suppose that EPCs might be essentially H-monocyte-derived CD14^+^ cells with variable expression of CD34, CD133, CD45, and KDR and angiogenesis capacity, as evidenced by Sieveking and colleagues [77]. Given the heterogeneous presence of EPC subpopulations in peripheral blood and absence of standardized criteria, we suggest considering the EPCs as “*putative cells.*” Their identification might be performed with a combination of several surface antigens. Other markers have been, indeed, detected, including platelet endothelial cell adhesion molecule-1 (CD31), CD146, von Willebrand factor (vWF), eNos, and E-selectin, C-kit, and CXCR4 (see Table 5) [13, 76]. Concerning methods for isolating and quantitatively or qualitatively evaluating these putative EPC cells, a large number of methodologies are disposable until now. However, immunohistochemistry or immunocytochemistry is principally used for quantifying EPCs in tissue samples [7, 76]. For circulating EPC evaluation, four different methods are available after their isolation from PBMCs: (1) cell culture of colony forming cells to reveal EPC features, that is, high proliferative potential, expression of endothelial markers, endothelial morphology, and formation of blood vessels in coculture experiments [7, 76]; (2) phenotypic EPC identification and enumeration by flow-cytometry analysis according to Duda protocol published in 2007 [78]; (3) quantitative real time PCR, which permits detecting and quantifying EPC specific markers in preenriched PBMC cell population [7, 76]; and (4) MCA method which includes magnetic (M) isolation of CD34^+^ cells from PBMCs, followed by a CD133^+^ immunocytochemical (CA) staining [7, 76]. To date, flow-cytometry and CFU assays are the two most used methods for EPC enumeration.

## 6. Conclusions and Recommendations: Standardized Criteria on EPC Investigations Are Imperative

The observations described above about the critical aspects on EPC cells point out the following considerations: (1) results of earlier studies on EPCs have to be reexamined; (2) the impact of these subpopulations has to be evaluated and considered only when future studies will be performed; (3) precise biological role or roles of several EPCs have to be clarified before their clinical application as both biomarkers to test cardiovascular health or candidates for cardiovascular cell therapy; (4) EPC definition using surface markers has to be reevaluated considering their heterogeneous origin and nature and probably performing not only flow-cytometry analysis but preferably a combination of other biomolecular assays.

In line with these considerations, recent advances in molecular EPC mechanisms highlight involvement of different growth factors and signaling pathways (i.e., VEGF, TGF-*β*, ROS, Wnt, Notch, and TLR-4 pathways) and microRNA in EPCs mobilization and differentiation into mature ECs [79, 80]. Future and intensive studies on the role of these molecules in EPC biology will be needed for improving or inducing vascular neoformation and angiogenesis in different CVD conditions. The common hope is in early overcoming various EPC problems and developing their real clinical applications, as biomarkers and regenerative cell agents. This might likely permit inducing and improving vascular regeneration under ischemic or other CVD events or provide a good substrate for vascular grafting, that is, bypass surgery and vascular reconstruction following aneurisms or traumatic injuries.

**Figure 5:**
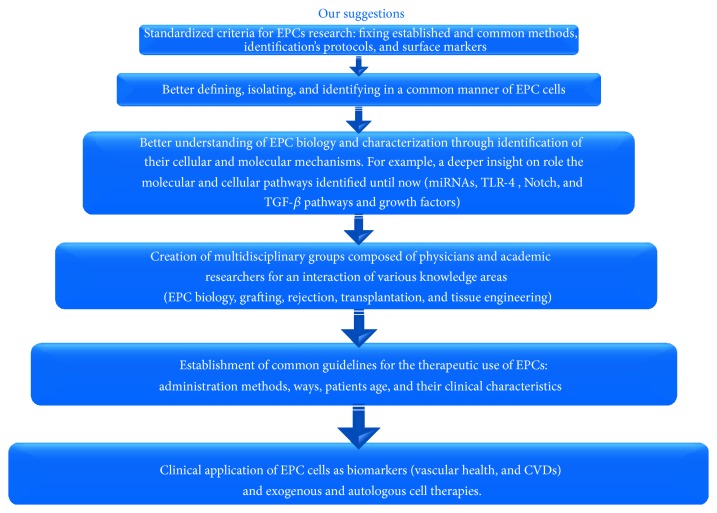
Our working hypothesis on the possible steps to perform to overcome the critical limitations and problems of EPC research and to develop real therapeutic applications.

In order to achieve this gold purpose, we suggest the following working hypothesis, as reported in Figure 5. Firstly, we underline that it is imperative to move versus a deep EPC characterization and precise definition, by performing future and further studies and establishing standardized criteria for EPC identification protocol and methods. This might really consent EPC defining and specifying functions. Probably, a combined and standardized analysis based on cytometric, transcriptomic, proteomic, and metabolomic evaluations might preferentially be needed for a definitive and true characterization of these cells, fixing standardized criteria. In addition, the development of an ideal EPC therapy and its clinical applications as CVD biomarkers might require the creation of interdisciplinary teams for fixing precise clinical elements of design and standardization. They might derive an intersection of investigations on EPC biology, tissue engineering, transplantation, grafting, rejection biology, clinical cardiovascular medicine, and device technology.

## Supplementary Material

The supplementary Material reports researched PubMed evidence on the role of EPC cells in CVDs.

## Figures and Tables

**Table 1:** Origins and sources of EPCs cells.

Stem and progenitor cells	Features and functions	References
*Hematopoietic stem or progenitor cells*		
Hematopoietic stem cells (HSCs)	Limited differentiation capacity compared to embryonic stem cells Commonly identified by the expression of CD34^+^ and CD133 cell surface antigens Clinically used for bone marrow transplantation in a variety of hematologic disorders Potentiality to differentiate into cardiac myocytes A subset of HSCs assume an endothelial phenotype promoting neovascularization by secreting proangiogenic growth factors and stimulating reendothelialization. These cells were named “endothelial progenitor cells” (EPC) The pattern of EPC surface markers includes CD133, VEGFR-2, CD34, Tie-1, Tie-2, CD146, c-Kit, and CXCR-4	[13, 15, 16]
H-myeloid cells	Mobilized from bone marrow CD14^+^/CD34^+^ myeloid cells coexpress endothelial markers, form tubelike structures *ex vivo,* and differentiate in endothelial cells incorporated in newly formed blood vessels Show a lower proliferation capacity than cord-blood-derived EPCs but have similar capacity to augment neovascularization in experimental models	[13, 15, 16]
H-mesenchymal stem cells (MSCs)	Limited differentiation capacity compared to embryonic stem cells Located in bone marrow and adipose tissues Transdifferentiate into functional cardiomyocytes and a variety of other cells Modulate immune responses	[13, 15, 16]

*Nonhematopoietic stem and progenitors cells (non-HSCs)*		
Fat tissue	Can be obtained in large quantities under local anesthesia with minimal discomfort Adipose tissue-derived stromal vascular cells lack both CD31 and CD34 markers and differentiate into ECs promoting angiogenesis	[13, 15–20]
Liver and intestine	Progenitor cells derived from transplanted liver and intestine contribute to neovascularization after hind limb ischemia it is still debated whether these incorporated progenitors are derived from vessel wall in the organ or they are tissue-resident progenitor cells of nonvascular origin	
Spleen	Can differentiate to give an “EPC phenotype” and modulate endothelial function or vascular remodelling	
Kidney	Pax-2^+^ cells displaying mesenchymal markers CD133^+^ cells derived from human renal carcinoma are able to differentiate into ECs and were found to be directly incorporated into neovessels	
Skeletal myoblasts	First cells to be injected into the ischemic myocardium as part of a cell-based strategy Determine improvements in left ventricular function but little evidence shows transdifferentiation into cardiomyocytes	
Blood vessel wall	MSCs cells also called pericytes or adventitial cells	

*Cord blood stem cells*		
	Greater plasticity than adult cells due to their prenatal origin Lacking evidence of pluripotency after *in vitro* expansion Cord blood contains a number of progenitor cell populations, including HSCs and MSCs Having not yet been investigated in a clinical setting	[13, 15, 16]

**Table 2:** Key players involved in modulating circulating EPC levels and functions.

Factors	Effects	References
*Factors and conditions associated with altered circulating EPCs levels*		
Aging	It determines a decrease in progenitor cells activity and mobilization	[26, 27]
Inflammation	Restricted acute inflammatory response stimulates EPCs mobilization while persistent chronic inflammatory stimuli have deleterious effects and result in decreased number of circulating mature and functional EPCs C-reactive protein (CRP) exerts direct inhibitory effects on EPC differentiation and survival. Proinflammatory TNF-*α* reduces EPCs number	[28, 29]
Oxidative stress	It reduces EPCs number, induces apoptosis, and reduces EPCs capacity of mobilization, migrating, and incorporating into vasculature	[28, 29]
Hypothyroidism	It decreases CD34^+^/CD133^+^/KDR^+^ EPCs	[31, 32]
Cardiovascular risk factors (smoking, diabetes, hypertension, lipid disorders, abdominal obesity, metabolic syndrome, etc.)	They influence the circulating levels of EPCs; precisely they reduce their levels	[30]
Hyperparathyroidism	It increases circulating EPCs levels	[31, 32]

*Physiological factors involved in EPCs mobilization*		
Gender	It upregulates VEGF and SDF-1 It modulates EPCs levels and cardiovascular risk profile, due to the beneficial effects of estrogens particularly in women The increase seems to be related to oestrogens levels	[33]
Pregnancy	It increases EPCs-derived colonies	[34]

*Drug therapies modulating circulating EPCs levels*		
Antihypertensive drugs		[35]
Calcium channel blockers (CCBs) nifedipine and barnidipine	They enhance EPC number and function	
Angiotensin II receptor blocker (ARB) telmisartan	It enhances EPC number and function	
Angiotensin converting enzyme (ACE) inhibitors	They improves clonogenic capacity	[35]
Cholesterol lowering medications		
Statines (atorvastatin, rosuvastatin)	They increase mobilization of EPCs and CD34^+^/CD117^+^, CD34^+^/CXCR4^+^	[35]
Antidiabetic medications		
Oral dipeptidyl peptidase-4 inhibitor (sitagliptin)	It increases number of circulating EPCs in patients with diabetes	[35]
Thiazolidinedione/metformin	They improve EPCs number and function	
Other drugs		
Estradiol	It improves capacity of neovascularization	[26]
PPAR-*γ* agonist	It increases EPCs migration	[26]
CXCR4 agonists	They stimulate SC mobilization	[26]
AMD3100 (plerixafor)	It increases CD34^+^, CD117^+^, and CD133^+^ cells	
POL6326		
Erythropoietin	It increases EPC and HSC levels	[24]
Nitroglycerin (chronic use)	It increases apoptosis and decreases phenotypic differentiation and migration	[26]
Granulocytes colony stimulating factor (G-CSF)	It induces SC mobilization by interruption of CXCR4/CXCL12, c-Kit/SCF, and VLA-4/VCAM-1 axis	[26]
Growth hormone	It reduces apoptosis	[26]
	It improves EPCs migratory capacity	

*Lifestyle modification and nutritional interventions*		
Red wine resveratrol, salvianolic acids, Gingko Biloba, ginsenoside, berberine, and puerarine.	They exert anti-inflammatory and antioxidant effects They enhance EPCs activity	[26]
Diet	It affects the number of circulating EPCs	[36, 37]
Dietary cocoa-derived flavonoids	They increase number of functional circulating angiogenic cells	[26]
Red ginseng extracts	They increase EPCs number	[26]
Physical exercise	It improves circulating EPCs levels. Prolonged 4-week exercise program improves EPCs functions. Maximal and endurance exercise influence the number of both EPCs and hematopoietic stem cells.	[26]

**Table 3:** EPCs therapeutic applications.

Preclinical studies in animal models		References
*Therapeutic approaches and effects*		
Animal models		
Rat model of myocardial infarction	Intravenous injection of *exvivo* expanded human CD34^+^ cells improves left ventricular function	[46]
Swine model of myocardial infarction	Catheter-based intramyocardial transplantation of EPCs leads to encouraging outcome	[47, 48]
Rat model of diabetes	Infusion of *invivo* expanded human EPCs augments conduction velocity and blood flow in sciatic nerve	[49]
Rat model of chronic cerebral ischemia	Injection of CD34^+^ HSC cells (including EPCs) improves neurological functions	[50]
*Macacus rhesus*	Skin autograft of CD34^+^ cells transfected with recombinant nonreplicative Herpes virus vector results in vector-gene expression and determines an increase in local angiogenesis	[51]

*Clinical trials in humans*		
Pathological CVD conditions		
Myocardial infarction	Endovenous administration of G-CSF as mobilizing factor for BM-derived progenitor cells improves left ventricular function	[52]
	Intracoronary infusion of BM-derived progenitor cells improves left ventricular function (TOPCARE-AMI and BOOST trials)	[53–55]
Diffuse coronary heart disease and angina pectoris	Transepicardial and transendocardial injection of unfractioned BM cells improve left ventricular function and physical capacity	[59–62]
Chronic limb ischemia	Direct administration of EPCs determines a reduced rate of limb's amputation at three-year follow-up	[63, 64]

*Autologous EPCs application*		
Pathological CVD conditions		
Chronic limb ischemia	Intramuscular injection of autologous BM-derived mononuclear cells containing 1% of CD34^+^ cells determines a local increase in endothelial markers (CD133 and VE-cadherin)	[65, 66]
Symptomatic coronary atherosclerosis	Administration of autologous EPCs expanded for four days in culture improves endothelial function and wall motion abnormalities, showing a benefic effect on the metabolism in the target area	[67]
Chronic limb ischemia	Intramuscular injection of autologous BM mononuclear cells improves local neovascularization (TACT study) and significantly lowers amputation rate at 3-year follow-up	[64]

**Table 4:** Methods and ways for the administration of EPCs cells.

First strategy: intravenous administration	BM-MSCs are transfused into the left ventricular cavity. Stem cells mainly reach the lungs, with significantly smaller amounts in the liver, heart, and spleen.	[52]

Second strategy: intracoronary infusion	Patients are infused with BM-progenitor cells using a balloon catheter after restoration of arterial patency	[53–55]

Third strategy: Transepicardial administration	Direct transepicardial injection of BMSCs can be performed, using a surgical thoracotomy into the border zone of the infarct	[59]

Third strategy: transendocardial administration	Catheter-based transendocardial injection of SCs using electromechanical voltage mapping to define tissue viability	[59]

**Table 5:** Surface markers used in EPC identifying.

Molecules	Biological features and relevance in EPC detection
CD34	105- to 120-kD transmembrane cell surface glycoprotein, selectively expressed (within human and murine hematopoietic systems) on stem and progenitor cells, and initially used by Asahara and colleagues for EPC identifying. It is not specific and expressed by mature endothelial cells as well as HSCs [76].

VEGFR2	A kinase insert domain receptor (KDR) or Flk-1, or CD309, suggested as further marker for identifying circulating EPC cells. It is expressed mainly on EC cells, and besides EPC cells, in low number, on osteoblasts, pancreatic duct cells, neuronal cells, and lung epithelial cells, even if the biological role in nonendothelial cells remains unclear. VEGFR2 has been shown to be a vital promoter of pathological neovascularization, including cancer and diabetic retinopathy, by making it a potential target in therapy of these diseases. However, neither of these markers is specific for EPCs, either alone or together. Vascular endothelial cells, expressing CD34 and VEGFR2, are not considered to be EPCs [76].

CD133	Also known as AC133. It is a marker of immature stem cells, proposed as the third marker for EPCs. Thus, EPCs have been identified as VEGFR-2^+^/CD133^+^/CD34^+^ cells. However, more than 99% of CD34^+^/KDR^+^/CD133^+^ triple positive cells also express CD45, which is a pan leukocyte marker, even if these cells are not able to give rise to EPCs capable of highly differentiating in endothelial cells. As such, CD45 expression on putative EPCs became a bone of contention [76].

CD31	Platelet endothelial cell adhesion molecule-1, also defined as PECAM [76].

CD146	S-endo, P1H12 antigen [76].

VWF	Von Willebrand factor [76].

eNos	Endothelial nitric oxide synthase [76].

E-selectin	Also known as CD62 antigen-like family member E (CD62E). Endothelial-leukocyte adhesion molecule-1 (ELAM-1), or leukocyte-endothelial cell adhesion molecule 2 (LECAM2), is a cell adhesion molecule expressed only on endothelial cells activated by cytokines [76].

C-kit	The protooncogene c-kit is a 145,000 Dalton transmembrane glycoprotein designed as CD117. This receptor tyrosine kinase and its ligand stem cell factor (SDF) mediate pleiotropic functions, including cell survival, differentiation, homing, migration, and proliferation as well as functional activation. It is present on the surface of cells of the mast cell and erythroid lineage as well as on multipotent stem and progenitor cells and megakaryocytes [76].

CXCR4	Also known as fusion or leukocyte-derived seven transmembrane-domain receptor (LESTR). It represents the receptor of SDF-1, highly expressed on the surface of CD34 positive cells [76].

UEA-I	Ulex europaeus lectin [76].
