# Supplementary material for: Are Endothelial Progenitor Cells the Real Solution for Cardiovascular Diseases? Focus on Controversies and Perspectives
Source: Biomed Res Int. 2015 Oct 5;2015:835934. doi: 10.1155/2015/835934 (PMC4609774; doi:10.1155/2015/835934)
Supplement: Supplementary file 1 — The supplementary Material reports researched PubMed evidence on the role of EPC cells in CVDs. [file 835934.f1.pdf]

Table 1 supplement Evidence of EPC cells in CVDs and other pathologies

STUDIES	PATHOLOGIES	VIVO/ VITRO	OBJECTIVES	CASES	SAMPLES	METHODOLOGY	RESULTS	BIOLOGICAL EFFECT	SURFACE MARKERS
Schmidt-Lucke C et al (1)	Atherosclerosis	Vivo	Circulating EPC provide an edogenous repair mechanism to counteract ongoing risk factor-induced endothelial injury	120 Patients	Peripheral blood	Flow citometry and immunochemestry	Reduced levels of circulating EPCs independently predict atherosclerotic disease progression	Prognostic effect	CD34+KDR+
Croce G et al. (2)	Hypercholesterolemia	Vivo	Evaluation whether or not changes in dietary habits, alone or in association with regular physical activity, where able to affect the number of circulating EPCs in patients with isolated hypercholesterolemi	38 Patients	Peripheral blood	Flow citometry and immunochemestry	Nonpharmacological treatment of hypercholesterolemia increases circulating endothelial progenitor cell population in adults	Therapeutic effect	DiI-acLDL and FITC-labeled Ulex-lectin
Vasa M et al. (3)	Stable coronary disease	Vivo	Circulating bone marrow-derived endothelial progenitor cells (EPCs) were shown to augment the neovascularization of ischemic tissue	15 Patients	Peripheral blood	Flow citometry and immunochemestry	Statin treatment in patients with stable CAD induced the increasing of circulating EPCs with enhanced functional activity	Therapeutic effect	CD34, CD133, and CD34/kinase insert domain receptor
Yao L. et al (4)	Atherosclerosis	Vivo (Animals Study)	Role of EPC in atherosclerotic plaque regression	Mice	Aortic samples	Immunoflorescence	EPC reduce the progression of atherosclerotic plaque	Therapeutic effect	Tie2-GFP(+)
Eirin et al. (5)	Essential Hypertension	Vivo	Relationship between EPC and essential hypertension in black patients	38 Patients	Peripheral blood, inferior vena cava and renal vein sample	Flow citometry and immunochemestry	Increased release of cytokines and inflammatory endothelial cells (IECs) in black EH patients may impair EPC reparative capacity and aggravate vascular damage, and accelerate hypertension-related complications	Prognostic effect	CD34+/KDR+ (EPC) and VAP-1+ (IEC)
Braitsch CM et al. (6)	Hypertensive heart disease	Vitro	Role of EPC in fibrosis associated with hypertension	Mice	Cardiac specimens	Immunochemestry	Aparticular phenotype of EPC, Tcf 21 positive, are involved in cardiac fibrosis associated with hypertension	Physiopathological mechanism	Wt1, Tbx18, and Tcf21
Ling L. et al. (7)	Acute myocardial infarction and type 2 diabetes mellitus	Vivo (Animal Study)	Relationship between type 2 diabetes and EPC in patient with myocardial infarction	62 Patients and mices	Peripheral blood	Flow citometry and immunochemestry	Bone marrow EPCs mobilization is delayed and reduced in diabetes	Prognostic effect	CD45−/low/CD34+/CD133+/KDR+
Caballero S et al (8)	Retinal vascular injury	Vivo (Animal Study)	Effect on retinal vascular homing of exogenous CD34(+) and CD14(+) progenitor cells using mouse models of chronic (streptozotocin [STZ]-induced diabetes) and acute (ischemia-reperfusion [I/R]) ocular vascular injury	Mice	Mice retinal fragments	Injection of Human CD34þ and CD14þ Peripheral Blood Mononuclear Cells and Murine Mesenchymal Stem Cells<br>Fluorescent Immunoistochemestry	Co-administration with MSCs or ex vivo fucosylation may enhance utility of CD34(+) cells in cell therapy for diabetic ocular conditions like macular ischemia and retinal nonperfusion	Therapeutic effect	CD34(+) and CD14(+)
Wan J et al (9)	Diabetic foot ulcer	Vivo (Animal Study)	Therapeutic effect of intramuscular transplantation of bone-marrow derived mesenchymal stem cells (BM-MSCs) in the leg for treatment of diabetic foot ulcers (DFUs) in rats	Wistar rats	Wound tissue samples	Immunohistochemistry,ELISA and RT-PCR	Intramuscular transplantation of BM-MSCs can significantly promote wound healing of DFUs in rats possibly as a result of increased expression of VEGF in the wound tissues	Therapeutic effect	CD31 and Ki-67
Alba AC et al. (10)	Heart failure	Vivo	Association between serial measurements of EPCs and functional capacity and outcomes in heart failure	156 Patients	Peripheral blood	Flow citometry and immunochemestry	Changes in circulating progenitor cells are associated with outcome in heart failure patients	Prognostic effect	CD34+, VEGFR2+ and/or CD133+
Padfield et al. (11)	Coronary artery disease	Ex Vivo	Correlation between EPC phenotype CD34(+)VEGFR-2(+)andCD34(+)VEGFR2(+)CD133(+) and severity of coronary artery disease	201 Patients	Peripheral blood	Flow citometry and immunochemestry	Traditional EPC populations, are not related to the extent of CAD or clinical outcome	Prognostic effect	C CD34(+)CD45(-)
Flammer AJ et al. (12)	Unstable Atherosclerosis	Vivo	Analysis of expression of osteoblastic marker osteocalcin + (OCN+) EPCs in patients with risk factors and a history of unstable atherosclerosis	282 Patients	Peripheral blood	Flow citometry and immunochemestry	Circulating OCN+ 'early' EPCs are strongly associated with unstable atherosclerosis	Prognostic effect	OCN+/CD133+/CD34-/KDR+
Kunz GA et al (13)	Coronary artery disease	Vivo	Role of EPCs in coronary artery disease.	122 Patients	Peripheral blood	Flow citometry and cell culture	Inverse relationship between circulating EPCs and CAD severity	Prognostic effect	CD34(+)CD45(-)
Chan KH et al (14)	Coronary disease	Vivo	Relationship between EPC populations and coronary epicardial and microvascular disease	31 Patients	Peripheral blood	Flow citometry and cell culture	The number and the function of late EPC is associated with CAD severity	Prognostic effect	CD34+/KDR +<br>CD34+/CD45+<br>CD34+/CD45-

D'Amario et al. (15)	Coronary bypass surgery	Vivo	Relantionship between ventricular remodelling and EPC after coronary bypass surgery	38 Patients	Peripheral blood	Flow citometry and immunochemestry	EPC represent a novel biomarker able to predict the evolution of ischemic cardiomyopathy following revascularization	Prognostic effect	CD34, CD45<br>CD90, CD105<br>IGF-1Rs
Taguchi A et al. (16)	Cerebral Ischemia	Vivo	Diminished numbers of circulating immature cells might impair such physiological and reparative processes, potentially contributing to cerebrovascular disfunction.	Patients	Peripheral blood	Flow citometry and immunochemestry	A possible contribution of circulating CD34- and CD-133 positive cells in maintenance of cerebral circulation in setting ischemic stress.	Prognostic effect	CD34-, CD133-, CD117-, and CD135+
Chu K et al. (17)	Cerebral Ischemia	Vivo/Vitro	Validation of the significance of colony-forming unit (CFU) and outgrowth cell yield in acute stroke.	Patients	Peripheral blood	Flow citometry, cell culture and immunochemestry	CFU number may thus represent an accumulated endothelial progenitor cell dysfunctional status, whereas outgrowth cell appearance may reflect the resilience of the systemic circulation to acute ischemic stress.	Prognostic effect	DiI-LDL, staining for UEA-1 lectin, CD31,<br>VE-cadherin, CD34, and KDR
Sobrino T et al. (18)	Cerebral Ischemia	Vivo/vitro	Evaluation of the prognostic value of EPC in acute ischemic stroke	Patients	Peripheral blood	Flow citometry, cell culture and immunochemestry	Increasing of circulating EPC after acute ischemic stroke is associated with good functional outcomes and reduced infarct growth.	Prognostic effect	
Bogoslovsky et al (19)	Cerebral Ischemia	Vivo	Correlation between EPC and ischemic lesion volume at baseline, lesion growth and final lesion volume	Patients	Peripheral blood	Flow citometry and immunochemistry<br>and MRI	Higher EPC levels were indicative of smaller volumes of acute lesion, final lesion, and lesion growth and may serve as a markers of acute phase stroke severity.	Prognostic effect	CD34+CD133+,<br>CD133+VEGFR2+, and CD34+ CD133+VEGFR2+
Vaturi et al. (20)	Bicuspid aortic valve	Vivo	Comparison of EPC level and function in patients with BAV with versus without valve dysfunction.	22 Patients	Peipheral blood	Flow citometry and immunotype characterization	Patients with BAV and significant valve dysfunction appear to have circulating EPCs with impaired functional properties	Physiopathological mechanism	CD133 and CD34
Shimoni S. et al (21)	Aortic regurgitation	Vivo	The aim of the present study was to assess the number and role of EPCs in patients with aortic valve regurgitation (AR).	31 Patients	Peipheral blood	Flow citometry and immunotype characterization	Patients with AR have fewer EPCs and late apoptotic EPCs	Physiopathological mechanism	CD34+/KDR+
Van Spyk et al. (22)	Abdominal aortic aneurysm	Vivo	CD34+ cells in patients with Abdominal Aortic Aneurysm (AAA) versus Peripheral Vascular Desease (PVD)	24 Patients	Peripheral blood	Flow citometry and immunochemestry	The AAA patients had a greater percentage of CD34+ cells compared with PVD patients AAA is a less severe vascular disease than PVD	Prognostic effect	CD34+
Schneider F et al (23)	Abdominal aortic aneurysm	Vitro	This study tested the hypothesis that bone marrow-derived MSCs (BM-MSCs) stabilizes AAAs in a rat model	Rat Fisher	Cell culture	Cell culture and immunochemestry	BM-MSCs exert a specialized function in arterial regeneration that transcends that of mature mesenchymal cells	Therapeutic effect	SM-alpha active positive cell
Li ZF et al (24)	Aneurysm	Vivo (Animal Study)	EPCs contribute to neointima formation and reendothelialization in rabbit elastase-induced aneurysm after flow diverter treatment.	Rabbit	Mice aortic fragment	Injection of Autologous EPC and<br>Fluorescent Immunoistochemestry	BM-derived EPCs participate in neointima formation in elastase-induced aneurysm after flow diverter treatment.	Therapeutic effect	Hoechst 33,342 and CFSE(carboxyfluorescein diacetate succinimidyl ester)
Grochot-Przeczek A et al (25)	Peripheral artery disease	Review	Role of EPC in peripheral artery ischemia						
Chan YH et al (26)	Prolongation of PR interval	Vivo		348 Patients	Peripheral blood	Flow citometry	PR prolongation is associated with endothelial dysfunction and evidence of endothelial repair activation in patients with high cardiovascular risk	Therapeutic effect	CD133(+)/KDR(+)
Oterino et al. (27)	Chronic Migraine	Vivo	Analysis expressing e-selectin, to assess endothelial activation and, therefore, endothelial dysfunction in migraine.	99 Patients	Peripheral blood	Flow citometry and immunochemestry	CD62E+ EPCs might be considered a marker for vascular damage in migraine patients	Prognostic effect	CD34+/KDR+<br>"early" EPCs those CD62E- EPCs, and "late" EPCs, CD62E+
Condorelli RA et al (28)	Arterial erectile disfunction	Vivo	Relationship between low pharmacological response to phosphodiesterase type 5 inhibitors and EPC	30 Patients	Peripheral blood	Flow citometry and immunochemestry	Non responder" patients showed a significant higher severity of penile arterial insufficiency and finally a significant higher level of endothelial apoptosis associated with higher serum concentrations of circulating late immunophenotype of endothelial progenitor cells.	Prognostic effect	CD45neg/CD34pos/CD144pos<br>CD45neg/CD144pos/Annexin Vpos
Rodrigo M et al. (29)	Sickle cell diseases	Vivo	Relationship between EPC and sickle cell disease	24 Patients	Peripheral blood	Flow citometry and immunochemestry	Low levels of EPc in patients with sickle cell disease increase the risk of cardiovascular disease	Prognostic effect	

References

1. Schmidt-Lucke C, Rossing L, Fichtlscherer S, et al. Reduced number of circulating endothelial progenitor cells predicts future cardiovascular events. Circulation. 2005;111:2981-2987.
2. Croce G, Passacquale G, Necozione S, et al. Nonpharmacological treatment of hypercholesterolemia increases circulating endothelial progenitor cell population in adults. Arterioscler Thromb Vasc Biol 2006; 26: e38-e39.
3. Vasa M, Fichtlscherer S, Adler K, et al. Increasse in circulating endothelial progenitor cells by statin therapy in patients with stable coronary artery disease. Circulation 2001;103:2885-2890.
4. Yao L, Heuser-Baker J, Herlea-Pana O, et al. Bone marrow endothelial progenitors augment atherosclerotic plaque regression in a mouse model of plasma lipid lowering. Stem Cells. 2012 Dec;30:2720-2731.
5. Eirin A, Zhu XY, Woollard JR, et al. Increased circulating inflammatory endothelial cells in blacks with essential hypertension. Hypertension. 2013 Sep;62:585-591.
6. Braitsch CM, Kanisicak O, van Berlo JH, et al. Differential expression of embryonic epicardial progenitor markers and localization of cardiac fibrosis in adult ischemic injury and hypertensive heart disease. J Mol Cell Cardiol. 2013;65:108-119.
7. Ling L, Shen Y, Wang K, et al. Worse clinical outcomes in acute myocardial infarction patients with type 2 diabetes mellitus: relevance to impaired endothelial progenitor cells mobilization. PLoS One. 2012;7(11).
8. Caballero S, Hazra S, Bhatwadekar A, et al. Circulating mononuclear progenitor cells: differential roles for subpopulations in repair of retinal vascular injury. Invest Ophthalmol Vis Sci. 2013 Apr 26;54:3000-3009.
9. Wan J, Cai Q, Liu Y. Effect of intramuscular bone marrow-derived mesenchymal stem cell transplantation in the leg for treatment of diabetic foot ulcers in rats. Nan Fang Yi Ke Da Xue Xue Bao. 2012;32:1730-1736
10. Alba AC, Lalonde SD, Rao V, Walter SD, Guyatt GH, Ross HJ. Changes in circulating progenitor cells are associated with outcome in heart failure patients: a longitudinal study. Can J Cardiol. 2013;29:1657-1664.
11. Padfield GJ, Tura-Ceide O, Freyer E, et al. Endothelial progenitor cells, atheroma burden and clinical outcome in patients with coronary artery disease. Heart. 2013;99:791-798.
12. Flammer AJ, Gössl M, Widmer RJ, et al. Osteocalcin positive CD133+/CD34-/KDR+ progenitor cells as an independent marker for unstable atherosclerosis. Eur Heart J. 2012;33:2963-2969.
13. Kunz G, Liang G, Cuculi F, et al. Circulating endothelial progenitor cells predict coronary artery disease severity. Am Heart J 2006;152:190-195
14. Chan KH, Simpson P, Yong AS, et al. The Relationship between Endothelial Progenitor Cell Population and Epicardial and Microvascular Coronary Disease- A Cellular, Angiographic and Physiologic Study. PloS One 2014;9:e 93980.
15. D'Amario D, Leone AM, Iaconelli A, et al. Growth Properties of Cardiac Stem Cells Are a Novel Biomarker of Patients' Outcome After Coronary Bypass Surgery. Circulation. 2013; 129:157-172.
16. Taguchi A, Matsuyama T, Moriwaki H, et al. Circulating CD34-positive cells provide an index of cerebrovascular function. Circulation. 2004;109:2972-2975.

17. Chu K, Jung KH, Lee ST et al. Circulating endothelial progenitors cells as a new marker of endothelial dysfunction or repair in cute stroke. Stroke 2008;39:1441-1447.
18. Sobrino T, Hurtado O, Moro MA, et al. The increase of circulating endothelial progenitor cells after acute ischemic stroke is associted with good outcome. Stroke 2007;38:2759-2764.
19. Bogoslovsky. Endothelial progenitor cells correlate with lesion volume and growth in acute stroke. Neurology. 2010;75:2059-2062.
20. Vaturi M, Perl L, Leshem-Lev D, et al. Circulating endothelial progenitor cells in patients with dysfunctional versus normally functioning congenitally bicuspid aortic valves. Am J Cardiol 2011; 108: 272 – 276.
21. Shimoni S, Bar I, Zilberman L, et al. Circulating progenitor and apoptotic progenitor cells in patients with aortic regurgitation. Circ J. 2013;77:764-771.
22. Van Spyk EN, Chun KC, Samadzadeh KM, Peters JH, Lee ES. Increased levels of CD34+ cells are associated in patients with abdominal aortic aneurysms compared with patients with peripheral vascular disease. J Surg Res. 2013;184:638-643.
23. Schneider F, Saucy F, de Blic R, et al. Bone marrow mesenchymal stem cells stabilize already-formed aortic aneurysms more efficiently than vascular smooth muscle cells in a rat model. Eur J Vasc Endovasc Surg. 2013;45:666-672.
24. Li ZF, Fang XG, Yang PF, et al. Endothelial progenitor cells contribute to neointima formation in rabbit elastase-induced aneurysm after flow diverter treatment. CNS Neurosci Ther. 2013;19:352-357.
25. Grochot-Przeczek A, Dulak J, Jozkowicz A. Therapeutic angiogenesis for revascularization in peripheral artery disease. Gene. 2013;525:220-228.
26. Chan YH, Siu CW, Yiu KH, et al. Prolongation of PR interval is associated with endothelial dysfunction and activation of vascular repair in high-risk cardiovascular patients. J Interv Card Electrophysiol. 2013;37:55-61.
27. Oterino A, Toriello M, Palacio E, et al. Analysis of endothelial precursor cells in chronic migraine: a case-control study. Cephalalgia. 2013;33:236-244.
28. Condorelli RA, Calogero AE, Favilla V, et al. Arterial erectile dysfunction: different severities of endothelial apoptosis between diabetic patients "responders" and "non responders" to sildenafil. Eur J Intern Med. 2013;24:234-240.
29. Rodrigo M, Mendelsohn L, Bereal-Williams C, et al. Circulating endothelial progenitor cells in adults with sickle cell disease. Pulm Circ. 2013;3:448-449.
